# A Pandemic Instrument can Optimize the Regime Complex for AMR by Striking a Balance between Centralization and Decentralization

**DOI:** 10.1017/jme.2022.76

**Published:** 2022

**Authors:** Isaac Weldon, Safaa Yaseen, Steven J. Hoffman

**Affiliations:** 1: YORK UNIVERSITY, TORONTO, ONTARIO, CANADA

**Keywords:** Regime Complex, Antimicrobial Resistance, Pandemic Instrument, Decentralization, Centralization

## Abstract

Global antimicrobial resistance (AMR) is currently governed by a decentralized regime complex composed of multiple institutions with overlapping and sometimes conflicting principles, norms, rules, and procedures. Such a decentralized regime complex provides certain advantages and disadvantages when compared to a centralized regime. A pandemic instrument can optimize the regime complex for AMR by leveraging the strengths of both centralization and decentralization. Existing climate treaties under the UNFCCC offer lessons for achieving this hybrid approach.

Antimicrobial resistance (AMR) is a complex, intersectoral, and long-enduring problem, with no single global institution set to comprehensively govern it. Instead, global efforts for AMR appear to be coalescing around what political science would call “the regime complex for antimicrobial resistance”; that is, a decentralized network of multiple institutions with overlapping and sometimes conflicting principles, norms, rules, and procedures.^1^ The regime complex for AMR includes United Nations (UN) organizations like the World Health Organization (WHO), Food and Agriculture Organization (FAO), the World Organization for Animal Health (WOAH; formerly OIE), and the United Nations Environmental Programme (UNEP), as well as many other governmental, commercial, and civil society stakeholders (Table 1).^2^ Such a decentralized regime complex provides certain advantages when compared to a centralized one, including promoting greater flexibility, adaptability, and resilience.^3^ However, it also presents several challenges for effective global governance, especially around navigating and uniting the growing number of international actors and institutions that each have their own distinct governance mandates and logics on the issue. Considering both the strengths and weaknesses posed by decentralization, great care must be taken about which aspects of AMR governance get centralized and which aspects do not – especially as the pandemic instrument provides an opportunity to deliver coordination and governance mechanisms for AMR.^4^ Fortunately, climate governance, which is similarly characterized by a decentralized regime complex, offers several lessons for how a pandemic instrument can achieve a hybrid of centralization and decentralization elements and benefit from the advantages of both.^5^

**Table 1:** The Regime Complex for AMR

Elemental Regime of Antimicrobial Resistance Complex	Principles	Norms	Rules	Proceures (multilateral bodies assemblies)
**Human Health Security**	State Sovereignty Health and security are linked Antimicrobials are essential to health security; AMR is a threat to health security Global health preparedness is possible through international cooperation Early warning systems are paramount for global health preparedness Economic health is inextricably tied to population health	States are responsible for Population Health and Health security Improving global health is a matter of protection against self-risk Information sharing Health interventions should minimize the impact on trade and travel	International Health Regulations (IHR) Resolutions of the World Health Assembly (WHA)	WHA IHR Annex 2
**Humanitarian Biomedicine**	Access to essential medicines including effective antimicrobials is a human right Human suffering can be alleviated with biomedical intervention Human suffering demands urgent and immediate response outside the framework of state sovereignty	State centricity Health for all Human right to health Improving global health is moral obligation	Universal Declaration of Human Rights Geneva Convention	United Nations Office for the Coordination of Humanitarian Affairs (OCHA) World Food Programme Médecine Sans Frontiers (MSF) International Committee of the Red Cross (ICRC)
**Animal Health**	State sovereignty Human health can be protected by protecting animal health Veterinary Services are a global public good	Animal welfare should be promoted and protected Information sharing Improving animal health is a matter of mitigating health risks for humans	World Organization for Animal Health (WOAH) Framework Rules and resolutions of the WOAH Sanitary and phytosanitary measures of the World Trade Organization (WTO) Terrestrial Animal Health Code Aquatic Animal Health Code Manual of Diagnostic Tests for Aquatic Animals Manual of Diagnostic Tests and Vaccines for Terrestrial Animals	WOAH (formerly OIE) Notification of disease outbreaks as outlined in rules and governing documents
**Agriculture**	State Sovereignty Food security and food justice demand that all people have physical and economic access to safe and nutrition food Antimicrobials are an important tool that can promote food security	Food systems should be sustainable, productive, and efficient Human, animal, plant life and health should be pursued simultaneously	Food and Agriculture Organization of the United Nations (FAO) constitution Resolutions of the FAO International Plant Protection Convention FAO Commission on Plant Genetic Resources and its Global System for the Conservation and Utilization of Plant Genetic Resources	FAO assembly
**Trade**	State sovereignty Intellectual Property rights promote progress and innovation, including for novel antimicrobials and therapies Liberalized trade contributes to health Most-favoured nation principle and non-discrimination in trade	Free trade Openness States have an enshrined right to protect human health Measures to reach other policy objectives should minimize impact on trade Private property rights	The Agreement on Trade-Related Aspects of Intellectual Property Rights (TRIPS) of the WTO The Agreement on the Application of Sanitary and Phytosanitary Measures (SPS) -The Agreement on Technical Barriers to Trade (TBT)	Ministerial Meetings WTO Dispute resolution mechanisms Compulsory Licensing
**Development**	Access to medicine including effective antibiotics is a human right Development depends upon access to certain health rights Development improves health and health security	Health for all Sustainable development and SDGs State sovereignty over natural resources	Convention on Biological Diversity (CBD) and its Nagoya Protocol on Access and Benefit Sharing United Nations Conference for Trade and Development BioTrade Initiative	CBD Conference of the Parties United Nations Development Programme
**Environment**	State Sovereignty Environmental health is essential for human health Common but differentiated responsibilities AMR in environment poses risks to environmental and human health	Sustainable Development Precautionary principles to potential degradation Benefits from biodiversity and environmental conservation should be shared Universal access to basic and common biological and plant genetic resources	1000s of Multilateral Agreements (MEAs) Some MEAs are focused on specific species, e.g., the 1946 Convention on Whaling and the 1979 Bonn Convention on the Conservation of Migratory Species Some MEAs seek to protect specific areas or ecosystems, such as the 1971 Ramsar Convention on Wetlands and the 1991 Madrid Protocol on the Antarctic. Some are about specific substances or types of substances, e.g., Basel, Rotterdam, and Stockholm Treaties on hazardous waste Convention on Biological Diversity and its Cartagena Biosafety Protocol Resolutions of UNEP	UNEP

Principles = beliefs of fact, causation, and rectitude

Norms = standards of behaviour defined in terms of rights and obligations

Rules = specific prescriptions or proscriptions for action

Procedures = prevailing practices for making and implementing collective choice^32^

This paper outlines the anatomy of the emerging regime complex for AMR. It then considers whether strategies applied in climate governance can be leveraged to improve the coherence of global AMR governance while harnessing the benefits offered by decentralization. More specifically, it argues that drawing on a hybrid approach and design elements from treaties within climate governance, namely, the Cancún and Paris Agreements, a pandemic instrument can leverage the responsiveness, flexibility, adaptability, and resilience of decentralization and the consistency, reliability, and efficiency of centralization.

## 1. The Anatomy of the Regime Complex for AMR

AMR is currently governed by a decentralized regime complex. Regime complexes are defined as partially overlapping and sometimes conflicting networks of three or more international regimes that relate to a common issue.^6^ For the multisectoral problem of AMR, it appears that there are at least seven international regimes coalescing around AMR governance, including the human health security, humanitarian biomedicine, animal health, agricultural, trade, development, environmental regimes (Table 1).

These seven elemental regimes, moreover, interact with at least one, but more often many other elemental regimes in the complex through bilateral and multilateral partnerships. For example, the human health, animal health, food, and environmental regimes interact through the WHO, FAO, WOAH, and UNEP’s quadripartite partnerships on AMR, the human health and agriculture regimes interact bilaterally through the WHO and FAO’s joint programmes on food standards and safety.^7^ The trade regime interacts with the human health regime, moreover, through the WHO, World Trade Organization (WTO), and World Intellectual Property Organization’s (WIPO) Trilateral Cooperation on Public Health, Intellectual Property, and Trade. It also interacts with the human health, animal health, agricultural regimes through various links among non-binding statements and the WTO’s Agreement on the Application of Sanitary and Phytosanitary Measures (SPS).^8^ Finally, there are also linkages among the trade, human health security, and environmental regimes on matters related to intellectual property rights, access to medicines, and environmental pollution.^9^

Many of the seven elemental regimes have a high degree of overlap in their memberships, but, crucially, there are important divergences in membership across the regime complex. For instance, the WHO and FAO are largely comprised of the same member states; but discrepancies in memberships exist across the trade, development, and biomedical humanitarianism regimes.

And finally, across the regime complex, there are several instances of overlapping and sometimes conflicting principles, rules, norms, and procedures. For example, both the human health security and the trade regimes believe that policy objectives such as promoting health should seek to minimize their impact on trade, while the principles of the human health, food, and animal health regimes all acknowledge the interdependencies among human, environmental, and animal health and the importance of One Health approaches. On the other hand, the trade regime’s principle that intellectual property rights improve health innovation, as well as the rules within the Trade Related Aspects of Intellectual Property Rights (TRIPs) that embody that principle, can often pose challenges for the development and humanitarian biomedicine regimes’ principle of access to medicine as human right.^10^

The following snapshot further elucidates the characteristics of the emerging regime complex for AMR (Table 1).

## 2. Strengths and Weaknesses of a Decentralized Regime Complex

The AMR governance landscape is currently populated by dozens of actors and institutions, each with their own mandates and initiatives (Table 1).^11^ As described above, these actors and institutions conflict in some instances, but there are also examples of cooperative synergies and partnerships emerging across them in others. The inherent challenges and opportunities present in the decentralized regime complex for AMR have led some to find the proliferation of actors as problematic for coherent governance, while others see it as an opportunity to mobilize and sustain action on multiple fronts.^12^ Indeed, the regime complex for AMR presents both disadvantages and advantages when compared to a comprehensive regime. On one hand, a more centralized regime promises to improve the coherence, consistency, reliability, and efficiency of global AMR governance, while also limiting free riding and gaming.^13^ On the other hand, decentralized regime complexes tend to provide more adaptability, flexibility, and resilience; enable more effective, equitable, and sustainable outcomes at multiple scales; and enhance innovation, learning, and trustworthiness among a greater number of participants.^14^

### Weaknesses of Decentralization

The most obvious challenge is that AMR’s decentralized governance structure makes it very difficult to parse through the many layers and different frontiers of global activity on the issue. In the absence of an overarching framework, such complexity poses challenges for understanding, tracking, and measuring progress on AMR.

Additionally, decentralization can pose equity challenges related to how priorities are set, decisions made, and resources distributed. In the absence of a global framework, wealthier states who invest in research and development for new antimicrobials and other technologies tend to do so according to their interests and needs. In other words, and as with many matters in global health, money sets the agenda especially in the absence of established global priorities.^15^ Moreover, as demonstrated by the ongoing inequitable global distribution of COVID-19 and MPox vaccines, new resources and countermeasures are not efficiently, effectively, or equitably distributed after discovery, which leads to inequitable and suboptimal use that prolongs global health emergencies.^16^

Without centralized coordination mechanisms to establish and sustain commitments to and investments for innovation, critical health infrastructure, and equitable distribution of medical countermeasures, AMR action will continue to skew toward the interests of high-income countries and not the long-term interests of the global public. This is especially problematic because AMR represents a weakest link challenge: the global response is only as strong as the weakest response in any given setting because it only takes the emergence and spread of one resistant microbe to undermine the efforts of all. This globally shared vulnerability requires centralized coordination and collaboration mechanisms to ensure equitable investments in global preparedness and prevention everywhere.

Without centralized coordination mechanisms to establish and sustain commitments to and investments for innovation, critical health infrastructure, and equitable distribution of medical countermeasures, AMR action will continue to skew toward the interests of high-income countries and not the long-term interests of the global public. This is especially problematic because AMR represents a weakest link challenge: the global response is only as strong as the weakest response in any given setting because it only takes the emergence and spread of one resistant microbe to undermine the efforts of all. This globally shared vulnerability requires centralized coordination and collaboration mechanisms to ensure equitable investments in global preparedness and prevention everywhere.

Beyond these challenges, overlapping and uncoordinated initiatives open the possibility for duplications and redundancies, while also leaving important gaps unaddressed — as is the current case with global leadership on crucial AMR initiatives such as surveillance.^17^ Finally, another important gap left unaddressed are mechanisms for global coordination to respond to resistant outbreaks that may emerge from the pandemic potential of AMR.

### Strengths of Decentralization

Despite these challenges, a decentralized regime provides several benefits.^18^ For example, by enabling bottom-up initiatives and creating more opportunities to develop cross-scale linkages, a decentralized regime complex can be more adaptable, resilient, and flexible compared to a centralized top-down regime. In the absence of top-down, command and control governance mandates, AMR initiatives are likely to be more adaptable to local challenges and leverage context specific knowledge from initiatives already in operation. Decentralization lets those with better knowledge of local needs and skills determine how best to address the issue and enables rules and norms to emerge organically from stakeholder led processes of social learning. A new top-down global framework for AMR, on the other hand, will likely suffer from the same equity and representational challenges that plague current global health governance structures.

Furthermore, the existence of redundancies could mean that there are extra fail-safes as well as multiple ongoing pathways to success.^19^ In other words, it could make a decentralized regime complex more thorough and resilient to failure, shocks, or shifts in global politics. Decentralization also enables greater opportunities for policy experiments, learning by doing, and trial and error initiatives across multiple locales, which can strengthen the evidence base of effective policy options while still making progress on the issue.

Finally, a decentralized regime complex can be more flexible than a centralized one. Flexibility allows countries to engage selectively with initiatives that are more relevant for their own domestic conditions. Flexibility also lets the rules bend without breaking, enhancing the sustainability of any international agreement. This could make cooperation around AMR more likely to work, since states usually engage with agreements and endeavors that they have faith in.^20^ It could also make cooperation more sustainable by letting states continue to cooperate even if they lag behind on some matters.^21^

## 3. Leveraging the Power of Centralization and Decentralization

One could argue that a decentralized regime complex, with its multiple centers of activity and diffuse initiatives, is the best option for generating and sustaining effective action on AMR quickly and meeting the need for more location specific knowledge and stakeholder driven solutions for AMR in the short run. To be sure, however, certain elements of global AMR require a more centralized governance structure to guide action in the long run. For instance, centralized mechanisms through the pandemic instrument could deliver much needed commitments and a global vision for global AMR efforts. It can also raise awareness based on a shared understanding of the issue and its urgency, enable the tracking of progress, distribute responsibilities and benefits equitably, and hold countries accountable to their promises.^22^

Considering the benefits offered by both, the best option for a pandemic instrument is to strike a balance between the responsiveness, flexibility, adaptability, and resilience of decentralization and the consistency, reliability, and efficiency provided by centralization. At the national level at least, studies have found that centralized and decentralized governance arrangements often co-exist.^23^ Somewhat paradoxically, centralization and decentralization are not exclusive and opposite ends of a spectrum, but are rather compatible and complementary ways of achieving institutional objectives.^24^

The same holds true for global governance arrangements for complex issues. The enormous regime complex for climate change, for example, has emerged with thousands of multilateral environmental agreements, organizations, and initiatives focused on addressing climate change, but recent efforts through the 2010 Cancún Agreements and, more recently, the 2015 Paris Agreement under the UNFCCC strike a balance between centralization and decentralization.^25^

The Cancún and Paris Agreements were negotiated against the backdrop of a growing regime complex for climate change and strategically leverage the benefits of decentralization under unified global frameworks to coordinate the global response to climate change.^26^ By using certain design elements, their architectures acknowledge that mitigating and adapting to the impacts of climate change require a hybrid of top-down centralization on some matters, with room for bottom-up initiatives on others.^27^

For example, the Paris Agreement contains a clear vision and a 2-degree Celsius global target that guides and gauges country behavior without necessarily prescribing it. This goal, in combination with the Paris Agreement’s reliance on individually determined contributions, lets countries determine how they will help meet the goal on their own terms. Put differently, the Paris Agreement provides top-down targets but relies on bottom-up implementation to achieve them.^28^ Meanwhile, the agreement’s established principles of universal and common but differentiated responsibilities, and its deference to centralized functions of the UNFCCC for matters such as surveillance, transparency, and accountability represent centralized mechanisms that complement its decentralized traits.^29^

Crucially, in relying on nationally determined, bottom-up initiatives as the main driver of global progress, the Cancún Agreements recognize that some countries require financial support to maximize their ability to address climate change and contribute to the universal global goal. In doing so, the Cancún Agreements established a Green Climate Fund, which, with its limitations notwithstanding, acts as a necessary financing mechanism to support less developed countries in implementing domestic climate action in this hybrid approach.^30^ The importance of the Green Climate Fund was reaffirmed in the Paris Agreement, which included mechanisms to enhance country contributions to it.

Finally, another key example of a hybrid mechanism embraced by both the Cancún and Paris Agreements are their commitments to the multi-stakeholder forum of the UNFCCC. The forum, as part of the UNFCCC’s Conference of Parties, provides a centralized mechanism and agenda for discussion and dialogue, but is inclusive enough such that many NGOs and interests groups can achieve observer status to attend, monitor, and question states-parties during the conference proceedings, thereby uniting a mix of centralized and decentralized elements.

A pandemic instrument could achieve a hybrid of centralized and decentralized elements by leveraging existing national action plans for AMR, while establishing a global goal against which to assess them and crystalizing a process for ratcheting up their ambition. Moreover, a combination of legally enshrined universal commitments that all countries must undertake, as well as a suite of common but differentiated responsibilities could further enshrine a balance of centralized and decentralized elements within the instrument.^31^ Other substantive items for pandemic treaty that would benefit from more centralization include:

1. Global information sharing and awareness raising mechanisms that are cognizant of location and scale, relevant knowledge, and evidence.
2. A standardized, consistent, and interoperable global surveillance system.
3. Transparency, accountability, and enforcement mechanisms.
4. A diverse and inclusive multi-stakeholder forum.
5. Global financing mechanisms, such as a global pooled fund, for global investments in infection prevention and control, and the innovation and distribution of medical countermeasures including antimicrobials, diagnostics, and alternative therapies. Alternative funding techniques could include a deposit mechanism or multi party-single closing type deals to leverage decentralized funds for AMR initiatives.
6. Global coordination for response to resistant outbreaks.

By adopting similar institutional design elements as the Paris Agreement, a pandemic instrument could provide much needed leadership on these specific issues in need of centralization while also being adequately flexible to encompass and permit location specific initiatives tailored to different national and local circumstances.

## Conclusion

Addressing AMR as a complex, cross sectoral issue requires coordination across the regime complex for AMR. But, counter-intuitively, the situation of decentralization that currently defines the AMR governance landscape presents some special advantages, and centralization is not necessarily the best option for all aspects of global AMR governance. Instead, a Pandemic Treaty should determine the value added by centralization, including consistency, enforceability, and reliability, and only include aspects of AMR governance that would appropriately benefit from these values, such as surveillance, global priorities, and funding mechanisms. It could then use design elements to benefit from the power of both centralization and decentralization, such as a universal global goal to guide nationally determined contributions; a combination of universal and common but differentiated responsibilities; an inclusive forum for priority setting, dialogue, and decision making; coordinated resource distribution systems; and a system to coordinate the global respond to resistant outbreaks. This hybrid approach could enable more centralized coordination on some activities but still let bottom up- and cross scale linkages thrive while benefiting from the adaptability, flexibility, and resilience provided by decentralization.

## Note

The authors thank the reviewers and editors for their helpful and constructive feedback. This article was written as part of IW’s Ph.D. Dissertation at York University, Toronto. IW thanks Anna Agathangelou, Clare Wenham, and James Orbinski for their insights, as well as the Canadian Institutes of Health Research for their financial support.
